# User Experience of Cognitive Behavioral Therapy Apps for Depression: An Analysis of App Functionality and User Reviews

**DOI:** 10.2196/10120

**Published:** 2018-06-06

**Authors:** Katarzyna Stawarz, Chris Preist, Debbie Tallon, Nicola Wiles, David Coyle

**Affiliations:** ^1^ Bristol Interaction Group Faculty of Engineering University of Bristol Bristol United Kingdom; ^2^ Centre for Academic Mental Health Population Health Sciences, Bristol Medical School University of Bristol Bristol United Kingdom; ^3^ National Institute for Health Research Bristol Biomedical Research Centre University Hospitals Bristol NHS Foundation Trust and University of Bristol Bristol United Kingdom; ^4^ School of Computer Science University College Dublin Dublin Ireland

**Keywords:** mental health, mobile apps, cognitive behavioral therapy, depression, user experience, mHealth

## Abstract

**Background:**

Hundreds of mental health apps are available to the general public. With increasing pressures on health care systems, they offer a potential way for people to support their mental health and well-being. However, although many are highly rated by users, few are evidence-based. Equally, our understanding of what makes apps engaging and valuable to users is limited.

**Objective:**

The aim of this paper was to analyze functionality and user opinions of mobile apps purporting to support cognitive behavioral therapy for depression and to explore key factors that have an impact on user experience and support engagement.

**Methods:**

We systematically identified apps described as being based on cognitive behavioral therapy for depression. We then conducted 2 studies. In the first, we analyzed the therapeutic functionality of apps. This corroborated existing work on apps’ fidelity to cognitive behavioral therapy theory, but we also extended prior work by examining features designed to support user engagement. Engagement features found in cognitive behavioral therapy apps for depression were compared with those found in a larger group of apps that support mental well-being in a more general sense. Our second study involved a more detailed examination of user experience, through a thematic analysis of publicly available user reviews of cognitive behavioral therapy apps for depression.

**Results:**

We identified 31 apps that purport to be based on cognitive behavioral therapy for depression. Functionality analysis (study 1) showed that they offered an eclectic mix of features, including many not based on cognitive behavioral therapy practice. Cognitive behavioral therapy apps used less varied engagement features compared with 253 other mental well-being apps. The analysis of 1287 user reviews of cognitive behavioral therapy apps for depression (study 2) showed that apps are used in a wide range of contexts, both replacing and augmenting therapy, and allowing users to play an active role in supporting their mental health and well-being. Users, including health professionals, valued and used apps that incorporated both core cognitive behavioral therapy and non-cognitive behavioral therapy elements, but concerns were also expressed regarding the unsupervised use of apps. Positivity was seen as important to engagement, for example, in the context of automatic thoughts, users expressed a preference to capture not just negative but also positive ones. Privacy, security, and trust were crucial to the user experience.

**Conclusions:**

Cognitive behavioral therapy apps for depression need to improve with respect to incorporating evidence-based cognitive behavioral therapy elements. Equally, a positive user experience is dependent on other design factors, including consideration of varying contexts of use. App designers should be able to clearly identify the therapeutic basis of their apps, but they should also draw on evidence-based strategies to support a positive and engaging user experience. The most effective apps are likely to strike a balance between evidence-based cognitive behavioral therapy strategies and evidence-based design strategies, including the possibility of eclectic therapeutic techniques.

## Introduction

### Background

Mental health difficulties are a leading cause of disability worldwide [1,2], with depression alone affecting 98.7 million people [2]. Responding to the urgent need to provide more people with access to effective treatments, substantial research has been undertaken on the use of technology to increase access to mental health treatment [3-8]. Much of this work has focused on the development and evaluation of computerized cognitive behavioral therapy (CBT) [5,8-13]. CBT incorporates both behavioral and cognitive aspects and provides a structured approach for recognizing and addressing negative thinking patterns and underlying beliefs [14]. Due to this structured approach, it lends itself well to being adapted to computerized platforms, both as self-directed [9] and therapist-guided [10] treatment.

More recently, mobile apps have provided an alternative to computerized CBT interventions. With 76% of UK and 81% of US adults owning a smartphone [15,16], there is a strong argument for the potential of apps to help in providing flexible access to mental health support [17,18]. Studies have been conducted focusing on the development of mobile apps to support mental health [19-28], and more detailed reviews and analyses of existing research are also available [4,29-31]. This work indicates the potential of appropriately designed apps and could drive future innovation in mental health apps to ultimately deliver large-scale impact on public health. However, given the openness of app stores to developers [32,33], challenges with regulating health apps [34], and the time it typically takes for evidence-based research to make its way into health care practice [35], it is unsurprising that current research is not always reflected in the apps available in app stores.

Recently, several papers have reviewed apps with the aim of assessing the extent to which they are grounded in theory, especially CBT [36,37], or to evaluate the extent of expert involvement [38,39]. They suggest that current apps tend to lack an evidence base [31,38] and often combine evidence-based features with other approaches not supported by research [37]. Furthermore, there does not seem to be any correlation between apps’ ratings and popularity and the presence of evidence-based features [37].

We agree that the lack of an evidence base in publicly available apps is a significant cause for concern. However, high ratings of apps defined as inconsistent with evidence [37] suggest that they might be important to users. The existence of these apps provides an important opportunity to investigate and understand factors that facilitate user engagement with mental health apps. Through app reviews submitted to app stores, people using these apps have provided a large body of data regarding their user experiences, context of use, and features they value. Previous work within the human-computer interaction (HCI) community has demonstrated the benefits of using public reviews to investigate user attitudes toward and experiences of existing apps [40-44]. Researchers have also analyzed user reviews of mood-tracking apps [44], looked at general use of health apps [45], or the types of health apps people with depression use [46]. Alongside efficacy, user experience and engagement are critical factors to the overall effectiveness of mental health technologies [47]; therefore, it is important to investigate what it is that the users themselves value.

### Objectives

This paper has 2 key aims: first, to systematically analyze the therapeutic elements and engagement approaches used in apps described as being based on CBT for depression; and second, to analyze publicly available user reviews of these apps to provide a more detailed understanding of the user experience and of what makes apps engaging and valuable to users.

In recent years, a number of important approaches have emerged for examining mobile health apps. For example, the Mobile App Rating Scale developed by Stoyanov et al provides a tool for assessing the quality of mobile health apps [48]. It includes a dedicated section on engagement that allows an assessor to score individual apps based on key engagement features. Chan et al provide a framework specifically for evaluating mobile mental health apps [49] that allows patients and mental health service providers to evaluate apps by their usefulness, usability, and integration with infrastructure. We view our approach as complementary, but distinct from these approaches.

Instead of providing detailed analysis of individual apps, we focused on the thematic analysis and synthesis of user perspectives on engagement and therapeutic features across a range of apps. Analyzing app reviews can provide insight on what end users find engaging in general and how the apps are used, and identify mismatches between what researchers believe to be important and what users actually find engaging. It can also help us to better understand why current apps are highly rated and leverage this understanding in the design of compelling, evidence-based apps going forward. As an approach, it can also offer distinct insights into recent app reviews that have incorporated traditional usability evaluations. For example, Huguet et al [36] applied Nielsen’s expert-led heuristic evaluation approach to assess the usability of CBT apps for depression. While usability helps to assess the degree to which users can easily—or with minimal training—use and understand the app, our approach offers complementary insights on *how* and *why* people use apps and the particular features which they find engaging or unengaging.

## Methods

### Data Collection

Our initial analysis of apps aimed to identify 2 key groups:

1. CBT apps for depression: apps that self-identify as implementing CBT to target depression.
2. Mental well-being apps: a broader group of apps that not only includes apps that aim to address mental health problems such as anxiety and depression, but also issues such as stress, worry, mood, or emotional well-being.

To limit the scope of our study, we deliberately excluded apps targeting more severe disorders such as bipolar disorder or less common disorders such as obsessive-compulsive disorder.

A detailed analysis of CBT apps for depression is the core focus of this paper. We consider both engagement features and therapeutic features (study 1) and user opinions (study 2). The set of apps addressing mental well-being more generally is not subjected to the same level of scrutiny as it is too large and too diverse in terms of therapeutic approaches applied. For these apps, we analyze engagement features only to allow a comparison with engagement approaches found in CBT apps for depression.

**Figure 1:**
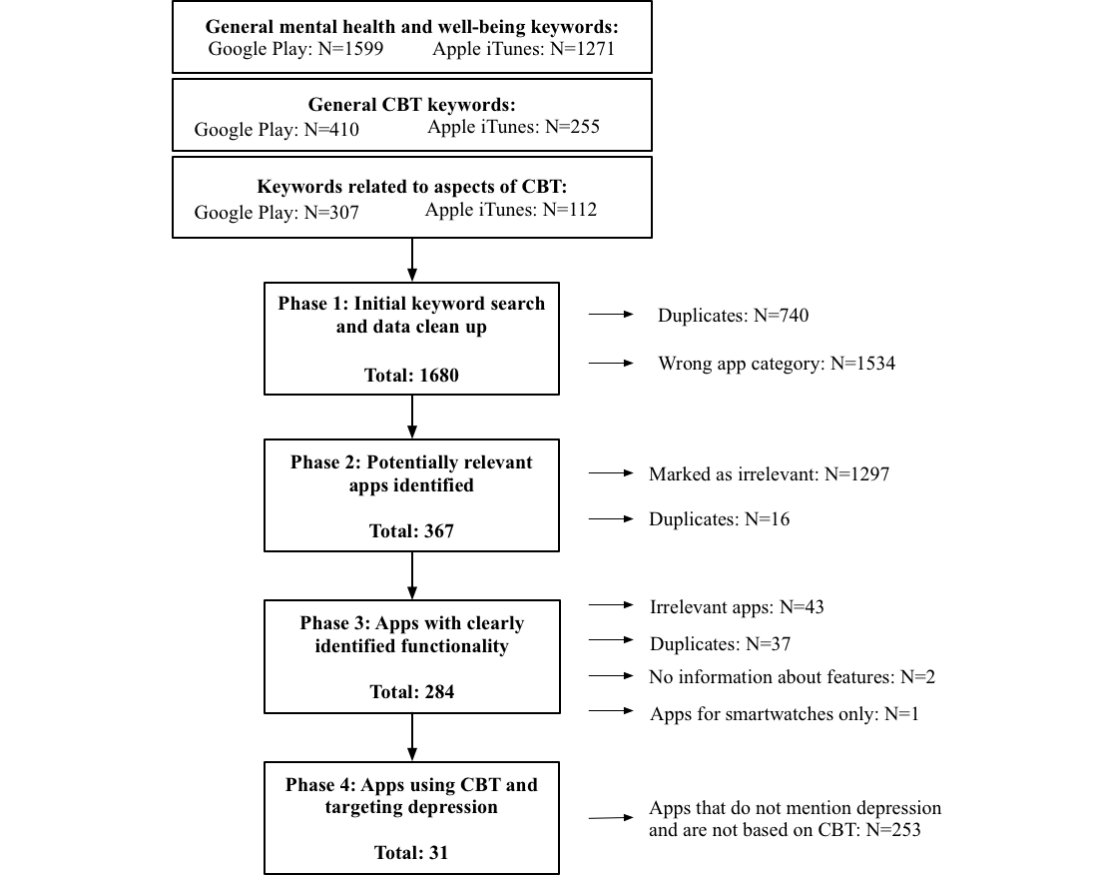
Data extraction and exclusion procedures. CBT: cognitive behavioral therapy.

Figure 1 illustrates the overall systematic process used to identify relevant apps.

#### Phase 1: Initial Keyword Search and Data Clean Up

We first defined the following groups of keywords:

1. General keywords related to mental health and well-being: “mental health,” “mental wellbeing,” “emotional wellbeing,” depression, anxiety, stress, mood, and emotions.
2. Keywords related to CBT: “cognitive behavioral therapy,” “cognitive behavioural therapy,” and CBT.
3. Keywords related to aspects of CBT: “activity diary,” “thought record,” “behavioral activation,” “behavioural activation,” “negative thoughts,” “core beliefs,” and “cognitive restructuring.”

We used scripts [50,51] to automatically download search results for each of these keywords separately from the UK version of Google Play and Apple’s App Store. The searches took place in January 2017. Recorded information included each app’s name, its short description (if available), detailed description, price, average rating, number of user ratings, developer’s details, and app store category. This resulted in 3954 apps (2316 apps from Google Play and 1638 from App Store).

We then used a custom script to combine the search results from each app store and remove duplicates. Finally, we automatically extracted apps belonging to the following app store categories (deemed to include relevant apps based on a manual check using the keyword “depression”): Health and Fitness, Medical, Lifestyle, Education, and Game Educational. We then combined the results and excluded duplicates of apps available for both platforms. At this point, 1680 unique apps remained.

#### Phase 2: Potentially Relevant Apps Identified

Next, we undertook the first manual screening. Following the approach used in the study by Shen et al [38], we manually reviewed the 1680 apps by examining each app’s title and short description. This allowed us to identify not relevant apps that would be excluded and potentially relevant apps that would be included in the next phase. We excluded the following types of apps:

1. Apps specifically addressing less common or more severe mental health disorders, for example, substance misuse, OCD; or other health conditions (eg, diabetes, chronic pain).
2. General health tracking apps and single purpose well-being apps, for example, for mindfulness meditation only.
3. Apps to support mental health professionals and students, and apps that require an access code (eg, that are part of a study, insurance plan, employer wellness scheme).
4. Apps not available in English.

A total of 1297 apps were marked as not relevant and further 16 turned out to be duplicates. This resulted in a set of 367 potentially relevant apps.

#### Phase 3: Apps With Clearly Identified Functionality

Next, we manually reviewed the full descriptions of all 367 potentially relevant apps. During this process, we identified 43 more apps that met the exclusion criteria described above; 2 apps that provided no information about their functionality; 1 app that was available for smartwatches only; and 37 duplicates. At the end of this phase, we had identified 284 apps.

#### Phase 4: Apps Using Cognitive Behavioral Therapy and Targeting Depression

The aim of this stage was to identify the final subset of apps that self-identified as (1) focusing on depression and (2) based on CBT. This resulted in 31 apps which we classified as *CBT apps for depression* and which we refer to as such throughout the paper. The remaining 253 apps were classified as *mental well-being apps*.

### Study 1: Functionality Analysis

The aim of this study was primarily to examine the functionality of the 31 CBT apps for depression. For each app, we recorded both engagement features and therapeutic features. We defined therapeutic features as functionality that aims to help users manage their mental health and well-being, and engagement features as functionality that encourages regular use, makes app content more appealing, and in general helps users to stay engaged with therapy or the app itself.

For each app, we recorded all features listed on its description page (eg, mood tracking, discussion forums, reminders, etc) or visible on screenshots; this approach has also been used in other app reviews [43]. We also noted mentions of expert involvement in app creation (health professionals, researchers, etc). Features were recorded by the first author and regularly reviewed and discussed with others.

Next, to assess whether the features of 31 CBT apps for depression reflect CBT practice, we asked 2 researchers (a clinical psychologist who is also an accredited CBT therapist, and an HCI researcher experienced in designing technologies to support CBT) to independently match them against a recognized CBT competence framework [14]. For each feature, they indicated (“yes” or “no”) whether it represented one of CBT competencies in relation to treatment for depression. Inter-rater agreement was 73% with raters disagreeing regarding 8 items, including 7 items where the disagreement was between a definitive answer (“yes” or “no”) and a “maybe.” Disagreements were resolved through a discussion, and the final categorization is available in Table 1.

Finally, we also undertook a brief analysis of the engagement features available in 253 mental well-being apps. A detailed review of the therapeutic features and approaches applied across this larger group was beyond the scope of this paper.

### Study 2: User Reviews Analysis

The analysis of publicly available app reviews has been successfully used in the past to investigate user attitudes toward existing apps and their feature requests [40-44]. We adopted this method to better understand users’ attitudes toward CBT apps for depression and which features they use and find the most important.

We used scripts [50,51] to automatically download all reviews for the 31 CBT apps for depression. If the app was available for both Android and iOS devices, we downloaded both sets of reviews. In total, we downloaded 2904 reviews of 24 apps (7 apps had 0 reviews). To identify reviews for the analysis, we followed the approach similar to the one used in the study by Stawarz et al [43]—first, the lead author manually assessed all reviews, recording their sentiment (positive, negative, neutral) and whether each mentioned at least 1 therapeutic feature; this was then discussed with other authors. Next, to qualitatively identify underlying themes, we used thematic analysis [52] to analyze the subset of reviews that mentioned at least 1 therapeutic feature. Coding was done by the first author and regularly discussed with others to allow for better familiarization with the data and reduce potential for bias. This iterative process led to codes gradually being merged into broader categories and researchers identifying overarching themes.

## Results

### Study 1: Functionality Analysis

Overall, within the set of 31 CBT apps for depression, we identified 26 therapeutic features and 10 engagement features; 4 additional engagement features were also available in the broader group of apps. App functionality is described in the following sections and features are summarized in Tables 1 and 2. Detailed information about the 31 CBT apps for depression, including their user ratings, available features, and expert involvement, are summarized in Multimedia Appendix 1.

#### Therapeutic Features of Cognitive Behavioral Therapy Apps for Depression

The most common features available in the 31 CBT apps for depression focused on dealing with negative automatic thoughts (48%, 15/31 apps) and negative thinking styles (29%, 9/31 apps), and provided examples of activities users could do to improve their mood (29%, 9/31 apps). They also allowed users to record thoughts and emotions (10%, 3/31 apps), schedule daily activities (10%, 3/31 apps), offered challenges and behavioral experiments (6%, 2/31 apps), or enabled goal setting (6%, 2/31 apps). The apps also offered several non-CBT features, including tools for writing and self-reflection, gratitude and affirmations, various tests and scales, or relaxation tracks. All features are summarized in Table 1.

#### Comparisons With Cognitive Behavioral Therapy Guidelines

Of 26 identified therapeutic features, 50% (13/26 features) reflected elements from the list of CBT competencies [14]. Overall, 90% of CBT apps for depression (28/31 apps) provided at least 1 CBT feature. 39% (12/31 apps) provided only 1 feature, while 29% (9/31 apps) provided only 2 CBT features; among these apps, features for dealing with automatic negative thoughts or negative thinking patterns were the most common. Moreover, 13% (4/31) of the apps provided 3 CBT features, 1 app (Cloud Clinic) had 4, and 1 app (Depression CBT Self-Help Guide) had 5. Finally, 1 app (MoodTools - Depression Aid) provided 8 CBT features. On the other hand, 10% (3/31) apps did not provide any CBT features at all. Instead, they mainly provided features such as positive self-talk and gratitude (7%, 2/31 apps), depression scales and self-assessment (7%, 2/31 apps), information about mental health and well-being in general (3%, 1/31 apps), and relaxation tracks (3%, 1/31 apps).

We also checked whether the presence of CBT features was associated with app ratings. Ratings were available for 24 apps with the average rating of 4.1 (on a 5-point scale), ranging from 2 for the Activity Diary app (which had 1 CBT feature) to 5 for MoodMaster Anti-Depression App (which had 2). There was no difference in average scores between apps with no CBT features (average score=4.1, min=4, max=4.2, N=3) and apps with at least 1 such feature (average score=4.1, min=2, max=5, N=21). The average rating for MoodTools, the most comprehensive app, was 4.3.

#### Expert Involvement

For each app, we recorded whether any experts were involved in the development process. Overall, 45% (14/31) of apps mentioned experts on their description page—health professionals (42%, 13/31 apps) and university researchers (7%, 2/31 apps). Apps with expert involvement provided between 0 and 8 CBT features (N=13, mean=2.3, mode=2), whereas the number of CBT features in apps that did not mention experts ranged from 0 to 5 (N=17, mean=1.6, mode=1). Health professionals were involved in the development of MoodTools, the app with the most CBT elements, and were also mentioned in the description of Self-Esteem Blackboard, an app that did not provide any features matching CBT elements. In contrast, 1 app with 3 CBT features (MoodSentry) was built by a patient who wanted to share the methods that worked for him.

There was also no link between the presence of expert involvement and user ratings. For example, MoodTools and What’s Up were the most rated apps with over 2000 user ratings each, and both had the average score of 4.3. However, the former offered 8 CBT features and experts were involved in its creation, whereas What’s Up offered only 2 such features and no experts were involved. On the other hand, Cloud Clinic received no ratings at all, despite expert involvement and presence of CBT features; both the lowest rated (Activity Diary) and the highest rated (MoodMaster Anti-Depression App) apps mentioned health professionals.

#### Engagement Features

For all mental health apps we identified, including 31 CBT apps for depression, we recorded details of features that aim to support user engagement with therapy or the app itself. They are summarized in Table 2.

Among the CBT apps for depression, 58% (18/31) of apps provided explicit engagement features. A quarter (26%, 8/31 apps) enabled sharing, including sharing with friends, family, and therapists. Graphs and charts to illustrate progress (13%, 4/31 apps), audio content (10%, 3/31 apps), and notification and prompts (10%, 3/31 apps) were also available. In addition, 2 of 31 apps (7%) offered personalization features and 2 other apps used gamification. Peer support and professional support can also drive engagement [47]; the former was available in 2 out of 31 (7%) and the latter in only 1 of 31 (3%) apps.

Among the broader group of mental well-being apps, 58.9% (149/253) provided engagement features. Graphs and charts were the most common (available in 16.6% (42/253) of apps) and were used to visualize users’ mood and progress. Reports and summaries that often accompanied graphs and charts were available in 1.9% (5/253) of apps. In addition, 9.1% of apps (2/253) provided various notifications and prompts—from reminders to record one’s mood or interact with the app in some way to weekly summary emails and the ability to set up medication reminders for antidepressants. Interactive content included video (9.5%, 24/253 apps), audio (7.1%, 18/253 apps), or game elements such as cartoon avatars, badges, or progress bars (2.4%, 6/253 apps). Customization and the ability to add own pictures and videos (2.4%, 6/253 apps each) helped to tailor the experience to users’ needs. Moreover, 1.2% (3/253) apps also used a Q&A format to make reading materials more engaging and to help users select the right activities. A total of 15 out of 253 apps (5.9%) enabled contact with therapists, including one-to-one chats, forums where health professionals can answer questions, or even the ability to schedule sessions.

#### Conclusions of Study 1

CBT apps for depression provided a mix of features. Even though all their descriptions mentioned CBT, only half of all features provided by apps reflected core competencies of CBT [14]. Moreover, most of the apps that did offer CBT features provided only 1 or 2 of them, and 3 apps did not provide any such features at all. This limited evidence base of apps is in line with existing research [31,37,38].

Available CBT features tended to be limited, focusing around mood tracking, recording thoughts and emotions, and dealing with negative thoughts. As a result, they often lacked elements of CBT used in high-intensity interventions for depression, such as addressing core beliefs [14]. The presence or absence of features grounded in CBT practice was not linked with expert involvement in app creation, which raises concerns regarding the responsibility of app creators who may be misleading potentially vulnerable users by mentioning CBT without actually providing it. There was also no clear link in terms of expert involvement and user ratings. However, our results highlighted high user ratings for all apps, regardless of whether they provided CBT features, which corroborates previous findings [37].

**Table 1:** Therapeutic features available in cognitive behavioral therapy (CBT) apps for depression (N=31), including information on whether each feature reflects CBT practice in relation to depression.

Therapeutic features	Definitions	CBT feature	Apps, n (%)
Dealing with negative automatic thoughts	Identifying and challenging specific negative automatic thoughts about self or the world	Yes	15 (48)
Addressing negative thinking styles	Identifying and challenging thinking styles and patterns; for example, catastrophizing, all-or-nothing thinking	Yes	9 (29)
Example activities	Example of pleasurable activities to do to improve one’s mood	Yes	9 (29)
Writing and self-reflection	Diaries and journals	No	6 (19)
Tracking mood	Tracking and annotating moods	Yes	5 (16)
Self-assessment	Tests and scales to assess one’s well-being	No	5 (16)
Gratitude and affirmations	Gratitude diary, examples of affirmations, ability to add affirmations, questions encouraging positive thinking about self	No	5 (16)
Information about CBT	Articles, blog posts, and other resources explaining cognitive behavioral therapy, its components, and how it works	Yes	4 (13)
Information about depression	Articles, blog posts, videos, and other resources explaining depression, its symptoms, how it works, and how to deal with it	Yes	3 (10)
Recording thoughts and emotions	Recording information about events, and thoughts and emotions that accompany them	Yes	3 (10)
Scheduling activities	Planning activities	Yes	3 (10)
Relaxation tracks	Calming music, sounds of nature, etc	No	3 (10)
General information about well-being	Articles, blog posts, videos, and other resources about mental health in general, health tips, well-being advice, nutrition, etc	No	3 (10)
Tracking anxiety and worries	Tracking anxiety incidents, worry lists	No	3 (10)
Recording and monitoring daily activities	Recording activities, matching activities with the calendar and mood information	Yes	2 (6)
Challenges and behavioral experiments	Tasks to complete to practice (new) coping skills	Yes	2 (6)
Setting goals	Setting up specific goals to works toward	Yes	2 (6)
Peer support	Ability to join forums or social networks, ask questions, and talk to others	No	2 (6)
Suicide prevention	Links to support services, ability to prepare a crisis plan	Yes	1 (3)
Challenging beliefs	Written exercises and examples of tasks to do to address one’s beliefs about the world and self	Yes	1 (3)
Breathing exercises	Written or recorded (audio or video) instructions for breathing exercises	No	1 (3)
Mindfulness	Mindfulness meditation tracks and written exercise instructions; excludes other types of meditation	No	1 (3)
Fun content	Games, jokes, and humorous content to provide distractions and improve one’s mood	No	1 (3)
Inspirational quotes	Quotes of famous people to provide motivation and lift one’s mood	No	1 (3)
Meditation	Guided meditation, topics to contemplate; excludes mindfulness	No	1 (3)
Physical exercise and yoga	Suggestions for specific exercises or yoga sessions	No	1 (3)

**Table 2:** Engagement features available in cognitive behavioral therapy (CBT) apps for depression (N=31) and other mental well-being apps (N=253).

Engagement features	CBT apps for depression (N=31), n (%)	Mental well-being apps (N=253), n (%)
Ability to share data directly from the app with others	8 (26)	28 (11.1)
Graphs and charts	4 (13)	42 (16.6)
Notifications and reminders	3 (10)	23 (9.1)
Audio content	3 (10)	18 (7.1)
Peer support	2 (7)	21 (8.3)
Customization	2 (7)	6 (2.4)
Games and gamification	2 (7)	6 (2.4)
Video content	1 (3)	24 (9.5)
Treatment program format (modules)	1 (3)	13 (5.1)
Ability to contact a therapist	1 (3)	15 (5.9)
Reports supporting graphs and charts	—	5 (1.9)
Ability to add pictures and videos	—	6 (2.4)
Chat with a bot	—	4 (1.6)
Q&A interface	—	3 (1.2)

Doherty et al [47] discuss interactive features, professional support, peer support, and customization as key strategies to facilitate engagement with therapy. Although a similar proportion of CBT apps for depression provided engagement features compared with the other mental well-being apps (58% vs 58.9%), these features were less varied. Interactive features such as video content, graphs, and charts; a bot interface that allows users to talk with a “virtual” therapist; or ability to add own pictures were more prevalent in the apps for mental well-being, although a bigger proportion of CBT apps offered audio content and gamification. Contact with peers or professionals was almost nonexistent in CBT apps for depression, possibly because peer support is not part of standard CBT and having an open forum would require moderation to reduce potential risks. However, CBT apps for depression offered more customization options, allowing users more options to adapt the features to their needs.

To better understand high app ratings and reasons why people use and value these apps, our second study focused on users’ attitudes toward app features and their experience of using CBT apps for depression.

### Study 2: User Reviews Analysis of Cognitive Behavioral Therapy Apps for Depression

Of the initially collected 2904 user reviews, 1287 reviews from 23 apps mentioned at least 1 therapeutic feature and therefore were included in the analysis; 91.61% (1179/1287) of these reviews were positive, 7.38% (95/1287) were negative, and 1.01% (13/1287) were neutral. The thematic analysis uncovered 4 key themes: different contexts of use; importance of privacy, security, and trust; importance of engagement features; and the attitudes toward therapeutic features not related to CBT. The themes are described below.

#### Context of Use of Cognitive Behavioral Therapy Apps for Depression

Reviews often mentioned context of use and how the apps fit into users’ lives. For 1 group of commenters, having a “pocket therapist” often meant using the app instead of therapy. It was often motivated by not being able to afford therapy or negative experiences in the past. The apps were also seen as simply better than regular therapy:

I just downloaded this for what’s probably obvious reasons—to try and get better, since I can’t afford therapy right now.

What’s Up

Another user said:

I have had depression for 3 years now and have found very little help from the [National Health Service]. These application from excel at life have helped me to relax and start to help myself. Couldn’t recommend more highly.

Depression CBT Self-Help Guide

In contrast, another group of commenters used the apps as an adjunct to treatment, alongside visits to a therapist. Users often commented on apps’ usefulness and how well they enhanced their treatment:

Great app. I always email my entries to myself so that I can print it out and share it with my psychiatrist.

Cognitive Diary CBT Self-Help

In some cases, users were using the app because it was the therapist who suggested that in the first place:

My therapist actually recommended this app and we trialed it in session, where it was really effective.

CBT Thought Record Diary

Regardless of why people used the apps, they generally appreciated their role in supporting their mental well-being. Comparisons to a “pocket therapist” or “therapy sessions at the tip of their fingers” were frequent:

Love this app, it’s like having a therapist in your pocket.

Depression CBT Self-Help Guide

Reviews were also written by therapists. They showed that sometimes clients were the ones who found the apps and integrated them into their work with the therapist, and at other times, therapists actively recommend apps to their clients:

I have several therapy clients who use this app rather than writing out a thought log.

iCBT

However, feedback was not always positive. Some users advised caution and warned that the apps should not be used without supervision, or even at all:

Can’t replace a therapist, especially when you’re first starting this therapy, but by directing the process, this is the next best thing.

Cognitive Diary CBT Self-Help

Another user mentioned:

Good design, bad idea. Apps shouldn’t be diagnosing medical conditions. Consider suggesting the user seek medical help PRIOR to using the app, and that the app should only be used in conjunction with treatment. Not as a precursor to treatment or as a reason to get treatment.

MoodTools

#### Importance of Privacy, Security, and Trust

As the apps play an important role in supporting users’ mental well-being, knowing that the service was reliable and their data were secure was important. However, this was not always the case—often apps were unreliable and losing data was experienced as “devastating”:

When the app upgraded, [it] erased ALL my examples in my Log. Anyone who practices CBT or has utilized the app [...] knows that this represents hours of work, and is practically irreplaceable. It is particularly bad news in a mental health app. [...] I was devastated about losing my data.

iCouch CBT

The analysis also revealed the importance of privacy and security, and users often mentioned them alongside therapeutic features. People appreciated the presence of password protection or security locks and demanded these features when they were not available:

Wish [I] could password protect diary. Some of my entries involve loved ones and I do not wish to cause them stress. I hide the app in my phone [and] my entries are written with little detail. Neither is ideal.

MoodTools

Another user said:

I used to keep a pen and pad as my diary. But people kept reading my personal thoughts and I felt very betrayed. Thanks to this app I can write how I feel in the midst of a situation. AND it has a PASSCODE to keep intruders out.

What’s Up

Users appreciated the discreet nature of the apps, and often compared them with paper worksheets, highlighting the privacy benefits the apps bring:

I like that it’s on my phone and therefore it’s always in my pocket, so if I’m stressing out over an issue I can pull it out and deal with the stress immediately. To people around me it just looks like I’m checking my phone, playing a game or writing an email.

MoodKit

In addition, this also meant that the users were more engaged:

One of my biggest struggles [during therapy] was actually doing the work and capturing things as they occurred. Having those tools in my pocket makes it convenient enough that I can do it any time I need to, and so I have been, and have benefited from it.

MoodKit

#### Importance of Engagement Features

Sharing was the most common engagement feature among CBT apps for depression and users appreciated the ability to share data with their therapist. Graphs and charts (more common among the wider set of mental well-being apps) and notifications were mentioned mostly in feature requests:

All I would improve is maybe reminder system. Like a reminder to update journal and to exercise mentally or physically.

MoodTools

One of the apps (What’s Up) provided a forum where users could talk and support each other. This feature polarized the users, who either loved or hated it:

I love the community! The encourage me, give me helpful advices and I have made some new friends!

What’s Up

Another user mentioned:

The forum is horrible. Just a bunch of teenagers giving each other advice on “cutting” techniques or which pills are best for suicide. Obviously not monitored. Very sad.

What’s Up

Comments also highlighted the importance of customization. Users were happy when the app was customizable, and demanded more flexibility when it was not:

It has a lot of helpful wording and allows you to add your own, to personalize it.

Cognitive Diary CBT Self-Help

Another user said:

There’s also not many customization options with statements. For instance, as an atheist, statements like “I can turn this over to God” are just not helpful to me, so I’d like to be able to hide them.

Worry Box

Therapists using an app with their clients also wanted the ability to customize it, with one observing:

I would have liked to have a way to list your own alternative coping statements along with the canned ones.

Worry Box

#### Attitudes Toward Therapeutic Features Not Based on Cognitive Behavioral Therapy

Users frequently commented on therapeutic features not based specifically on CBT, especially relaxation tracks and meditation. Commenters also appreciated examples of activities they could do to improve their mood and the ability to track anxiety episodes and worry (although the majority of those comments referred to Worry Box—an app designed specifically to deal with these issues). Writing in a diary was also often mentioned, although it was not always clear whether the mentions referred to simple journaling or a structured thought diary. The majority of the users valued having both therapeutic features based on CBT and other approaches:

For a free app that provides cbt logs, meditation, relaxation training, the ability to track some of you depression symptoms & provide suggestions to get you up and moving—it’s a well designed little app.

Depression CBT Self-Help Guide

However, some indicated that the use of CBT was important because of its strong scientific or evidence base and did not like more eclectic apps:

This app is presented as a straightforward cognitive therapy app, but is riddled with pseudo-spiritual New Age nonsense. Avoid.

Depression CBT Self-Help Guide

Some therapists who used an app with their clients were positive about the integration of these different types of features in a single app, with one commenting:

I was looking for an app to use with clients in my clinical work and I really like this one. Having both the relaxation tracks and a way to challenge unhelpful thoughts was great.

Worry Box

Furthermore, the main “criticism” voiced by many users, together with associated feature and customization requests, was the absence of positivity:

It stresses ONLY negative feelings. I believe that focusing a person solely on their darker aspects only reinforces those aspects of their daily outlook.

MoodTools

Another user mentioned:

I really didn’t like how I could only write unhelpful thoughts. I do have positive thoughts too. I want to write those down.

Thought Diary

#### Conclusions of Study 2

The analysis of user reviews showed that users appreciated all therapeutic features, including both the ones based on CBT as well as on other approaches, and so did professionals using the apps, which can explain the lack of correlation between high ratings and presence of evidence-based features reported in other studies (eg, [36,37]). Moreover, users wanted those other, non-CBT features, especially the ones focusing on more positive experiences.

Apps were often mentioned in the context of therapy—as “pocket therapists,” they often replaced or augmented therapy, allowing users to take an active role in supporting their own mental well-being. Their discreet nature was particularly important. However, reliance on the app and the type of data users entered meant that privacy, security, and trust were important. And when that trust was violated, for example, when the app lost the data, it had serious consequences, leaving users devastated.

## Discussion

### Principal Findings

We presented 2 studies that investigated factors that make mental health apps engaging: in study 1, we examined engagement features available in CBT apps for depression, and the relationship between the presence of CBT features and expert involvement and the app ratings; in study 2, we thematically analyzed publicly available user reviews to understand user experience and contexts of use. Our results show that apps are used in a wide range of contexts, both replacing and augmenting therapy, and allowing users to take an active role in supporting their mental health and well-being. Users, including health professionals, valued and used apps that incorporated both core CBT and non-CBT elements, but concerns were also expressed regarding the unsupervised use of apps. Positivity was seen as important to engagement, for example, in the context of automatic thoughts, users expressed a preference to capture not just negative but also positive ones. Privacy, security, and trust were crucial to the user experience. We discuss these findings below.

#### Integration Into Different Therapeutic Practices

The results showed that apps were used as part of different therapeutic practices—as part of therapy as well as a tool for self-management. A mix of different features and varying contexts of use provides a challenge to app developers, but at the same time opens up opportunities for integrating apps into therapeutic practice. The prevalence of features related to key CBT concepts (eg, negative automatic thoughts, mood tracking) that do not necessarily require input from a therapist and are often covered in self-directed computerized CBT suggests that apps may, in particular, be a useful addition to low-intensity CBT. They may be particularly effective in facilitating engagement with CBT homework (which often requires recording thoughts and emotions, planning activities, or tracking mood), which is a desirable outcome as regular engagement with such CBT exercises increases the effectiveness of therapy [53,54]. Moreover, the results show that apps have important advantages with regard to integration into practice—they are always at hand, are more private and discreet than paper worksheets, and enable easy sharing of data with the therapist. Taking a smartphone out when in a shopping queue or on a bus is perfectly “normal” behavior, that is, it is familiar to the individual and (usually) acceptable to those around them. Therefore, people can integrate such exercises into everyday life and reduce concerns about “getting caught” completing therapy tasks in public [55].

#### Engagement With Therapy

Engagement with therapy or therapeutic content can be achieved through different means, including interactive features, peer or professional support, or customization [47]. Each of these approaches was found to some degree across the apps we examined, although CBT apps for depression lacked features enabling contact with others and more interactive app-based engagement techniques such as bots or the ability to upload own content. Study 2 findings suggest that reminders to use the app, in particular, are something that users would value. However, care must be taken when designing such reminders to ensure they are effective and not annoying [56]. As with other health apps (eg, menstruation trackers [57]), they also need to ensure privacy—it was clear from the user comments that people shared their phones with others and thus would want to keep private the fact that they use an app to support mental health. In exploring this area, many lessons could be drawn from existing literature on behavior change [21], health promotion [7], or medication adherence apps [43].

It is common in CBT interventions that therapists provide personalized examples or tailor exercises based on a client’s needs [14]. Study 1 showed that personalization features were more prevalent among CBT apps for depression, and study 2 showed that both users and therapists appreciated the ability to customize apps, which reflects this personalized nature of therapy. This raises questions of how best to support customization of exercises. One option is for the customization to be done by the therapist, and appropriate content uploaded into the client’s app. A second option involves a therapist and client working together to customize the app, for example, to set specific exercises for the client to complete between therapeutic sessions. Alternatively, the user (outside a therapeutic relationship) could make such modifications themselves, which some apps already allow. However, when customizations have the potential to impact the therapeutic effectiveness of an app, putting such abilities in the hands of the user would require responsibility on the part of the app designer, to help ensure that such customizations are therapeutically appropriate. The means of implementing this is an important subject for future research.

#### Building on an Evidence Base From Multiple Fields

The analysis of our data suggests that many end users, including therapists, valued flexibility in the use of therapeutic approaches. Similar to the finding of the study by Kertz et al [37], we found that many apps mixed both CBT and non-CBT features. Some users felt this was inappropriate, particularly when they thought the latter were not evidence-based. However, they were a minority and a number of commenters expressed a desire for features beyond standard CBT, such as the ability to record positive emotions. Although positive logs are used in CBT, the apps tend to focus on negative automatic thoughts and negative thinking styles. Moreover, some therapists seemed to approve the combination of CBT techniques with, for example, relaxation audio. Many users find such a blend to be more engaging, even if not faithful to a core CBT model [14]. Given the importance of engagement in achieving effective outcomes, this raises important questions for the design of mental health apps and potential benefits of non-CBT features in CBT-focused apps.

Prior research on eclecticism emphasizes that it should not be construed as antitheoretical. Rather, eclectic approaches should be guided by some integrating framework that gives coherence to the overall therapeutic process. Within the context of traditional, face-to-face mental health interventions, several such frameworks have emerged [58]. Debate regarding the efficacy of eclecticism versus adherence to a core intervention model has a long history and is the subject of ongoing research [58-60]. A detailed discussion of this literature is beyond the scope of this paper. However, better understanding of how eclecticism can be effectively supported through apps, either with or without therapist support, is an important subject for future research. Promising initial work has been carried out by Mohr et al [26], who developed IntelliCare—an eclectic suite of apps that provide a wide range of features (their Thought Challenger was among the set of 31 CBT apps we identified). In addition, the most comprehensive app in our set, MoodTools, has been developed by health professionals, and is a good example of how different approaches can be blended. The potential benefits of combining CBT with approaches from Positive Psychology [61,62] is another clear message from our work. Moreover, although it is crucial that apps should draw on clinical theory, this alone is not enough. They also need to be based on research grounded in other domains such as HCI, which provide evidence of effective approaches to building desirable and engaging computer systems [23,47].

#### Responsibility Toward the Users

With potential users expressing different needs, and therefore using the apps in different ways, the above discussion raises the question of the responsibility held by app designers or sellers. Regulating health apps is a challenge [34], raising several ethical and practical issues. Should designers take responsibility for the ways in which their app is used? Should they somehow police it? It is clear that some users felt that certain apps should not be used by someone untrained in CBT or outside a therapeutic relationship. Assuming this view is correct, how can designers ensure that their app is not used inappropriately? Is there potential for the identification and warning of inappropriate use patterns? Alternatively, it may be appropriate to block unsupervised use of apps, for example, through access codes provided by professional therapists to their clients, as used by the Pesky gNATs app [63] and others.

This responsibility also extends to the stability and reliability of the app. While an app failure in general can be inconvenient and annoying, it can have serious consequences in the context of mental health apps—someone who has come to rely on an app for emotional support can find a failure “devastating.” This is also an issue with other health-related apps, for example, app updates can lead to a loss of scheduled reminders from medication adherence apps [43]. Therefore, reliability and backward compatibility of upgrades should be tested more thoroughly than for other types of apps. New providers entering the market should think seriously whether they can take on such responsibility.

This responsibility arguably applies to the app stores as well. They already encourage thorough testing of the apps before launch [32,33], routinely examine the technical implementation of apps, and block apps that do not meet the required technical standards. If an app is available in the health section, and offers mental health support, should the app stores be expected to police its quality from a health or clinical perspective? Perhaps such app listings should be required to explicitly state which health professionals (if any) were involved in its design, what evidence (if any) is available for the techniques it offers, and also provide guidance as to which contexts of use the developers feel are appropriate. Guidelines for evaluating health apps [64], objective app guides [65], and dedicated health app stores (eg, the one curated by the UK National Health Service [66]) are a good first step, but they do not solve the potential issues with apps widely available in commercial app stores.

### Limitations

To assess whether the features of the 31 CBT apps for depression reflect CBT practice, we compared them with a recognized CBT Competence Framework [14]. It is important to note that this competence framework was developed for assessing competency of face-to-face therapists, not for CBT apps. Given the current absence of a widely recognized competence framework for CBT apps for depression, we believe this was the best approach.

Our focus on app stores means that the results reflect apps that are commonly available, rather than apps that are the current state-of-the-art apps in research. We believe that by exploring the experience of current users, our work complements other research and can help to inform future designs. Similarly, given the focus on user reviews in study 2, there is an obvious potential for selection bias toward extreme ratings and positive reviews [67,68]. However, we believe that focusing on specific features and context of use allowed us to reduce this bias while still providing relevant insights. Although the reviews were coded by 1 author, the coding and the data were regularly discussed with the rest of the team to ensure everyone is familiar with the data, which is an acceptable approach in qualitative analysis [52,69,70]. Overall, although not definitive, our approach allowed access to user experience data that would otherwise be extremely difficult to obtain. It provided data on experience across a large number of different apps, which people have used over different durations, as part of their daily life. In-depth studies of individual apps do provide stronger data, but not the same breath of coverage. They also prescribe how a user in a study is expected to use the app, and so provide less insight into the development of in-the-wild usage patterns in response to personal situations.

### Conclusions and Future Work

Drawing from a rich pool of public app reviews, our research shows that users use apps alongside and instead of therapy, with the same app being used in both contexts. It also suggests that features not considered evidence-based may be key to facilitating user engagement. The challenges and opportunities we have identified open up new avenues for research. Future work should explore approaches to integrating apps into different therapeutic practices, facilitating engagement, finding a balance between drawing from clinical and design research, and exploring different approaches toward responsibility and accountability, and the role of app stores as gatekeepers.

## Appendix

Multimedia Appendix 1 Details of 31 CBT apps for depression.

## Abbreviations

CBT: cognitive behavioral therapy
HCI: human-computer interaction
